# Differential microRNAs and metabolites in the breast milk of mothers with adverse childhood experiences

**DOI:** 10.1038/s41398-025-03491-4

**Published:** 2025-10-06

**Authors:** Weronika Tomaszewska, Anna Apanasewicz, Magdalena Gomółka, Maja Matyas, Patrycja Rojek, Marek Szołtysik, Magdalena Babiszewska-Aksamit, Bartlomiej Gielniewski, Bartosz Wojtas, Anna Ziomkiewicz, Ali Jawaid

**Affiliations:** 1 Translational Neuropsychiatry Research Group (TREND Lab), Lukasiewicz Research Network- PORT Polish Center for Technology Development, Wroclaw, Poland; 2 Laboratory for Translational Research in Neuropsychiatric Disorders (TREND Lab), BRAINCITY: Center of Excellence for Neural Plasticity and Brain Disorders, Nencki Institute of Experimental Biology, Warsaw, Poland; 3 Department of Anthropology, Hirszfeld Institute of Immunology and Experimental Therapy, Polish Academy of Sciences, Wroclaw, Poland; 4 Laboratory of Anthropology, Institute of Zoology and Biomedical Research, Jagiellonian University, Krakow, Poland; 5 Department of Functional Food Products Development, Wroclaw University of Environmental and Life Sciences, Wroclaw, Poland; 6 Department of Medical Biology, Medical University of Warsaw, Warsaw, Poland; 7 Laboratory of Sequencing, Nencki Institute of Experimental Biology, Warsaw, Poland

**Keywords:** Human behaviour, Predictive markers

## Abstract

Adverse childhood experiences (ACE) can strongly impact the physical and mental health of individuals. Recent evidence further suggests that children born to mothers with a history of ACE are at an increased risk for behavioral and metabolic perturbations. In this study, we investigated the impact of maternal ACE on small RNAs and fatty acids (FAs) in the breast milk from a cohort of Polish mothers (*n* = 103) and ascertained their association with early temperament of their children. Small RNA sequencing followed by qPCR assays were performed to compare small RNAs in the milk from lactating mothers with high vs. low ACE. Additionally, milk from mothers with high vs. low ACE were compared on the short-, middle-, and long-chain FAs content. Our study revealed distinct microRNA and FA signatures of ACE in human breast milk; with increased expression of miR-142-3p, miR-142-5p, and miR-223-3p and reduced levels of middle chain FAs (MCFAs) in the breast milk of mothers with high ACE. Furthermore, a positive correlation was observed between the milk expression of miR-142-3p, miR-142-5p, and miR-223-3p and the ACE score in the mothers. Finally, milk expression of miR-142-5p and MCFAs correlated with infant temperament at the age of 5 and 12 months. The observed associations were not confounded by symptoms indicative of postpartum depression in the mothers. In conclusion, this study newly reveals changes in milk miRNAs and FAs as signatures of ACE in humans and highlights their potential as predictors of intergenerational transmission of the effects of ACE.

## Introduction

Recent research has increasingly recognized that many adult-onset mental and physical diseases have their roots in the cumulative effects of childhood adversity [1, 2]. Adverse childhood experiences (ACE), which include exposure to emotional, physical or sexual abuse, neglect, parental separation or demise, or living through conflicts, can pervasively impair brain and body functions. Consequently, ACE heightens the risk of various mental and physical health impairments in adulthood ranging from depression, anxiety, and post-traumatic stress disorder (PTSD) to diabetes mellitus, dyslipidemia, and cancer [3–7].

According to the World Health Organization (WHO), nearly 3 in 4 children experience some sort of adversity before the age of 4 years [8]. The exact prevalence of different forms of ACE varies based on the study design and population. However, the lowest reported prevalence of ACE is around 20%, which aligns with the WHO reported global prevalence of childhood malnutrition [9, 10]. Notably, the E-risk (Environmental Risk Longitudinal Twin) study, which followed 1116 twin pairs from birth to age 18 in the UK, found that 1 in 3 participants experienced victimization, neglect, or abuse [11, 12]. Similarly, a cross-sectional assessment of 6027 children and adolescents between the age of 7 and 17 in China identified exposure to at least one significant traumatic event in 34.3% of the participants [13]. Importantly, sex-specific patterns have been reported for both the likelihood of ACE, as well as their long-term impact. Females are more likely to experience certain forms of ACE, such as sexual abuse, and also have a greater risk of depression and PTSD after ACE [12, 14].

The biological understanding of the long-term sequelae of ACE has recently been revolutionized by the discovery that the effects of ACE are transmissible across generations. Children of ACE-exposed parents are more susceptible to depression, post-traumatic stress disorder (PTSD), and metabolic syndrome [15, 16]. Changes in germline non-coding RNAs (ncRNAs) and metabolite-responsive receptors have been identified as major vectors for such intergenerational effects of ACE. Notably, ACE in the form of unpredictable maternal separation and unpredictable maternal stress (MSUS) has been associated with wide-ranging behavioral and metabolic deficits in both the ACE-exposed mice, as well as their progeny [17, 18]. These effects are accompanied by significant overlapping ncRNAs changes in the sperm of ACE-exposed mice, as well as the serum and brain of the offspring. Furthermore, injecting RNA from the sperm of adult ACE-exposed mice into the 1-cell embryo from naive mice recapitulates the intergenerational transcriptomic and phenotypic changes associated with ACE [17]. Beyond the role of ncRNAs, overlapping changes in the circulating metabolome following ACE in mice have also been implicated in intergenerational transmission of phenotypes. Notably, injecting serum from ACE-exposed mice to naive mice altered their sperm receptor signaling and transmitted specific phenotypes to their offspring [18]. These studies highlight the importance of circulating ncRNAs and metabolites in preservation and propagation of the effects of ACE.

While the intergenerational transmission of the effects of ACE can occur through both the male and female germ cells, two additional mechanisms of intergenerational transmission warrant a closer examination in females; passage of the offspring through the birth canal and lactation [19]. Lactation, in particular, could be a potential pathway for the intergenerational transmission of the effects of ACE. Breast milk is rich in microRNAs (miRNAs) and fatty acids (FAs), both of which have been previously associated with intergenerational transmission of symptoms the effects of AC [17, 18, 20]. Furthermore, breast milk miRNAs are known to regulate the expression and functions of several genes critical for neurodevelopment [21–23]. Similarly, FAs in breast milk have been linked to the cognitive and temperamental development of the offspring [24]. Finally, numerous studies have shown that maternal health factors, such as age, body weight [25–28], infections [29], and psychological stress [30, 31] can alter the bioactive compounds in the milk.

To investigate the role of breast milk miRNAs and FAs in potential intergenerational transmission of the effects of ACE, we conducted a multi-modality prospective study of a mother-child cohort. The children analyzed in the study were exclusively breastfed for the first five months of age. We first examined miRNAs and FAs in the breast milk of mothers exposed to ACE using unbiased approaches followed by confirmation for selected miRNAs. Additionally, we investigated the association between the miRNA and FA profiles in the breast milk of ACE-exposed mothers and the temperament of their offspring over the first year of life.

## Material and methods

### Study design and ethical considerations

The study group comprised 103 mother-child dyads from the city of Wroclaw, Poland. The recruitment of dyads took place between January 2017 to September 2018 based on stringent inclusion criteria. The inclusion criteria for mothers required them to be at least 18 years old at delivery, to have delivered by singleton vaginal birth that followed an uncomplicated pregnancy course, with complete abstinence from alcohol and tobacco use during pregnancy and lactation, and no use of steroids during lactation. The inclusion criteria for the children included near or full-term birth (at least 37 gestational weeks), appropriate birth weight to gestational age (at least 2500 g), being exclusively breastfed for at least five months after birth and absence of any congenital diseases.

**Fig. 1:**
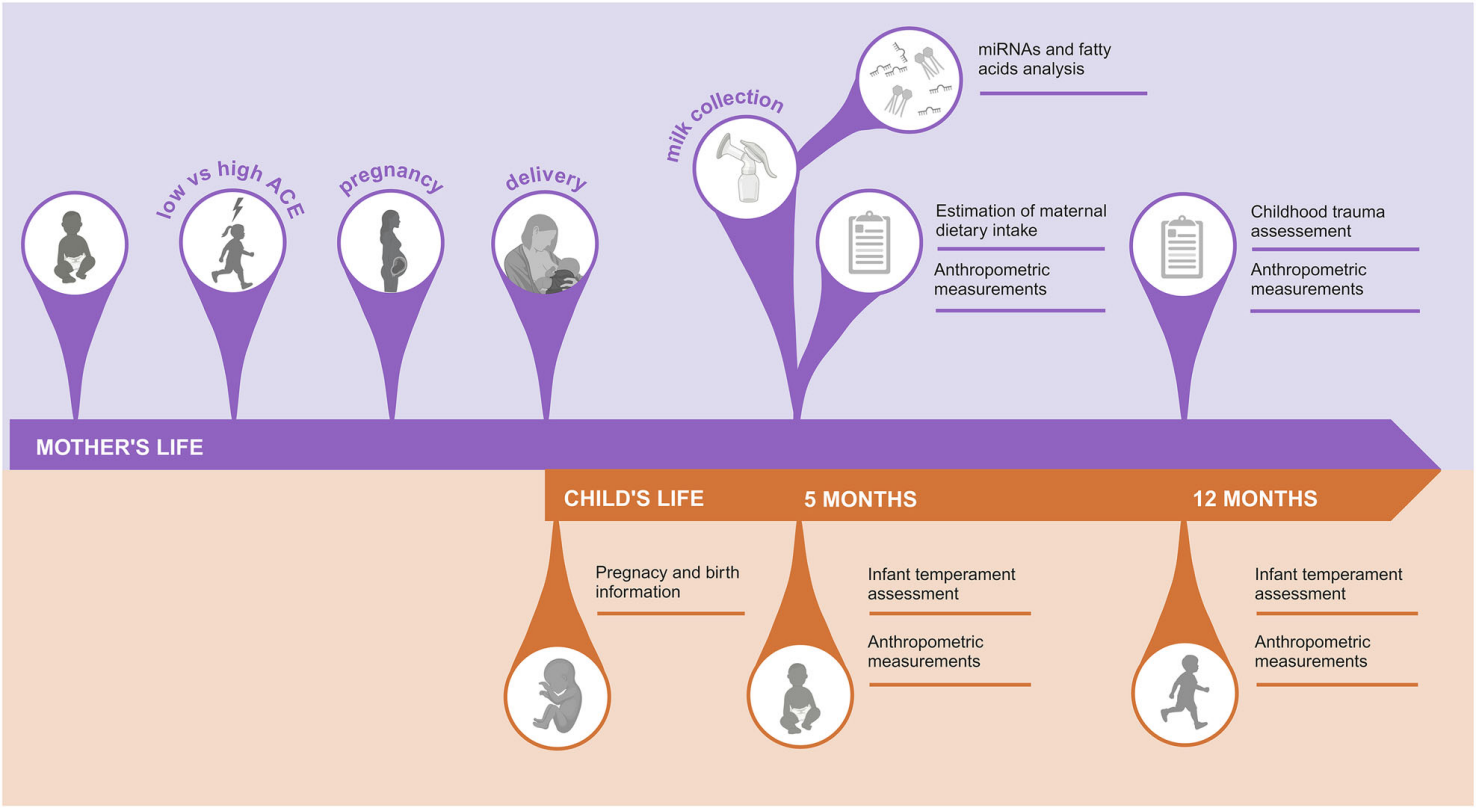
Graphical summary of the study procedures.

The mother-child dyads were assessed at three different time points: at birth, and then at 5 months and 1 year postpartum. Pregnancy and birth outcomes were obtained from the birth health records of the mother-child dyads. During the visit at 5 months postpartum, information on maternal and infant health, maternal dietary intake, and infant temperament was acquired. Additionally, trained assistants collected anthropometric measurements from both mothers and infants, along with breast milk samples. At the 12 months postpartum visit, further assessments of maternal and child health status, infant temperament, as well as repeat anthropometric measurements of the dyads were conducted. We also examined ACE in mothers during this visit to mitigate any potential influence of interview-induced stress on milk parameters and quality of maternal care (Fig. 1).

All participants received information about the purpose and course of the study and gave informed written consent on behalf of themselves and their children at the time of the first visit. The participants were appraised of the option to resign from the study at any stage without any legal or financial consequences as per the stipulated principles of the Helsinki declaration. The study protocol was approved by the Bioethical Committee of the Lower Silesian Medical Chamber in Wroclaw (approval identification number 1/NT/2016 from 10.02.2016).

### Anthropometric measurements

The anthropometric measurements of mothers and children were recorded three times for cross-validation. The measurements for mothers included assessments of body weight using the Tanita SC-240 MA scale (Tanita, Poland) (accuracy of 0.1 kg) and height with a stadiometer (GMP, Swiss) (accuracy of 0.1 cm). Furthermore, body mass index (BMI) was calculated for the mothers using the formula BMI = body weight [kg]/ (body height [m]^2^). For the children, body weight was measured with an analog hospital scale (accuracy of 0.1 kg), length with the Seca measuring board model 417 (Seca, Germany) (accuracy of 0.1 cm), and head circumference using an anthropometric measuring tape (Fiberglass tape, Thailand) (with an accuracy of 0.1 cm). Data related to children’s birth weight, length, and head circumference were collected from their health records.

### Psychometric assessments

The history and severity of ACE was assessed in the mothers via the Polish version of the Early Life Stress Questionnaire (ELSQ) [32] derived from the Child Abuse and Trauma Scale [33]. The ELSQ includes 19 events associated with subjective feeling of intense traumatic stress: including exposures to natural disasters, bullying, long-lasting illness, domestic violence, sexual abuse, and isolation from the family. The mothers were requested to indicate the exposures in the first 12 years of their life [32]. Furthermore, when the children were 5 months old, mothers’ postpartum depression (PPD) risk was assessed based on the Polish version [34] of the Edinburgh Postpartum Depression Scale (EPDS) [35]. It includes 10 items assessing symptoms of PPD on a 4-point (0–3) scale [36]. A score of 13 or more on EDPS indicates significantly increased risk of PPD [37].

Mothers’ assessment of their children’s behavior was ascertained through the Polish version [38] of the Revised Infant Behavior Questionnaire (IBQ-R) [39]. This questionnaire examines the infant temperament in 14 behavioral traits (approach, vocal reactivity, high-intensity pleasure, smiling & laughter, activity level, perceptual sensitivity, sadness, distress to limitation, fear, falling reactivity, low-intensity pleasure, cuddliness, duration of orienting and soothability). These traits were clustered into three main behavioral factors based on factor analysis as previously described by Dragan et al.: 1) surgency/extraversion (high-intensity pleasure, approach, soothability, smiling & laughter, cuddliness, and vocal reactivity), 2) negative affectivity (sadness, distress to limitation, activity, and falling reactivity), and 3) orienting regulation (fear, low-intensity pleasure, perceptual sensitivity, and duration of orienting). Overall, IBQ-R includes 191 questions that can be scored 1–7 with the additional option of skipping individual questions by selecting the statement “This does not apply to my child” [38].

### Maternal food intake

The women were asked to complete a daily food intake record including meals, drinks and dietary supplements for three days, two of which were weekdays and one day from the weekend. The daily food intake was then averaged out from these three days. The daily intake of macronutrients (energy density, FAs, proteins, and carbohydrates) was assessed using the software Dieta 5.D developed by the National Public Health Institute in Poland.

### Breast milk collection

The mothers received detailed instructions for breast milk collection by a trained research technician blinded to the study design. Milk samples were self-collected using the Medela Symphony medical breast pump with disposable one-day set (Medela AG, Switzerland), and sterilized bottle at the laboratory. The samples were collected one hour after the second feeding episode (between 9 am and 11 am) to avoid diurnal rhythmical changes in breast milk composition [40, 41]. The participants were permitted to choose between the left or right breast for milk collection as there is no evidence that the human milk composition differs between the two breasts [42]. Furthermore, the participants were instructed to pump the milk from the breast until the breast was empty [43]. The collected samples were then divided into smaller aliquots for long-term storage and further analysis. A 20 ml fresh sample was used for FAs analysis, whereas the rest of the aliquots were stored at −80 °C.

### Breast milk composition analysis

Overall, macronutrient (protein, fat, carbohydrates) composition and the energy density of the milk samples were analyzed by mid infrared transmission spectroscopy using MIRIS Human Milk Analyzer (MIRIS, Sweden) according to the producer’s instruction. Subsequently, 15 ml of the fresh milk samples were used for FAs analysis. First, the total lipid fraction was extracted using chloroform: methanol (2:1 v/v) followed by Nitrogen-induced desiccation [44, 45]. Following this, 1 μl samples of the lipid fraction were analyzed using gas chromatography (Agilent Technologies 5973). The concentration of FAs (g per 100 g total fat) was determined based on the obtained peak areas and calibrated to the standard curves for the analyzed FAs. These estimated concentrations were used to further calculate the total quantity (g) of given FAs and classes of FAs per 100 ml of milk. Finally, data on individual FAs was grouped into medium-chain FAs (MCFAs), long-chain saturated FAs (LCSFAs), long-chain monounsaturated fatty acids (MUFA) and long-chain polyunsaturated fatty acids (PUFAs) (Supplementary Table 1).

### RNA extraction from milk

RNA extraction from the milk samples was performed using miRNeasy serum/plasma Kit, according to the manufacturer’s protocol (Qiagen, Germany). This kit allows the best quality and quantity of miRNA from the lipid fraction of milk [46]. Briefly, 800 μl of whole milk was centrifuged for 20 min at 4 °C at 700 g. After centrifugation, 200 μ of milk lipid fraction was isolated for RNA extraction. Mean yield of RNA extracted (based on Nanodrop measurements) was 157 ng/μl. Quality varied from A260/280 1.64–2.03 (mean 1.90).

### RNA sequencing and pathway analysis

RNA integrity was evaluated with Agilent 2100 Bioanalyzer, using RNA 6000 Pico Kit (Agilent Technologies, California, United States). Following confirmation of RNA integrity, miRNA libraries were prepared with Qiaseq miRNA Library Kit according to the manufacturer’s protocol (Qiagen, Germany). Libraries were assessed with Agilent 2100 Bioanalyzer using Agilent High Sensitivity DNA Kit (Agilent Technologies, California, United States). Libraries were quantified using a Quantus fluorometer and QuantiFluor double stranded DNA System (Promega, Wisconsin, USA). Libraries were single-end sequenced (75 bp) on NovaSeq 6000 (Illumina, California, USA).

Primary miRNA bioinformatic analysis was performed using, Qiagen’s data analysis center (Qiagen GeneGlobe Data Analysis Center). Unique molecular identifiers (UMIs) were quantified in five steps: 1) calibration of miRBase entries, 2) trimming of adapters and low-quality bases, 3) identification of UMIs, 4) sequence alignment using the MiRBase V21 and piRNABank databases, and 5) quantification of unique molecules. Samples with miRNA read counts less than 200,000 were excluded from further analysis. The sequencing data was corrected for multiple comparisons using a 10% false discovery rate (FDR). PCA (principal component analysis) was performed to visualize the distribution of the data and exclusion of batch effects. For secondary bioinformatics analysis, differential expression analysis was performed using a DESeq2 Bioconductor’s package. Non-expressed and low-expressed miRNAs were discarded in the pre-filtering step. The ggplot2 package was used for visualization of miRNA expression differences between the groups.

### qPCR confirmation of selected miRNAs

All miRNAs that proved to have a statistically significant difference based on the sequencing results at 10% FDR (p-adj <0.1) were further quantified using reverse transcription-quantitative polymerase chain reaction (RT-qPCR). Reverse transcription was performed on purified RNA samples with miRCURY LNA RT Kit (Qiagen, Germany) followed by RT–qPCR using the miRCURY LNA SYBR Green PCR Kit (Qiagen, Germany) on the CFX Opus 96 Real-Time PCR System (Bio-Rad Laboratories, California, United States). The selected miRNAs were probed by specific miRCURY LNA miRNA primers for: hsa-miR-142-3p, hsa-miR-142-5p and hsa-miR-223-3p (Qiagen, Germany). The endogenous control hsa-miR-146b-5p was chosen for normalization of qPCR data based on comparable expression between the very high vs. low ACE groups on sequencing (Supplementary Table 5) and literature review [47, 48]. All samples were run in triplicates under the following cycling conditions: 95 °C for 2 min, 40 cycles of 10 s at 95 °C, 1 min at 56 °C, followed by a melting curve with gradual temperature increase from 60–95 °C. Melting curve analyses were performed to confirm amplification of single products for each primer.

### Statistical analysis

All comparisons between the two main groups (high ACE vs. low ACE), such as epidemiological parameters, psychometric assessments, as well as expression of selected miRNAs were performed using independent sample t-test or Mann-Whitney test based on the data normality. Comparisons between three groups (ACE 0-1; ACE 2; ACE ≥ 3) were performed using Kruskal-Wallis test with Dunn’s multiple comparisons test. Furthermore, separate ANCOVA models were built to examine differences between breast milk FAs according to maternal ACE level (low-high) and infant sex (boy-girl) as binomial independent variables and maternal BMI and fat intake as continuous independent co-variates. Spearman rank correlation test was performed to ascertain the correlations between expression of milk miRNAs with mothers’ ACE scores, as well as milk miRNAs and FAs with infant temperamental scores. Statistical analyses were performed with GraphPad Prism version 10.4.2 (GraphPad Software, Massachusetts, United States) and R version 4.3.2 with supplemental packages (R Core Team, 2021). The graphs were prepared with GraphPad Prism version 10.4.2.

## Results

### Demographics and clinical characteristics of mother-child dyads

The ELSQ score of the mothers (*n* = 103) ranged between 0 and 11 traumatic events by the age of 12. Only 14.6% of the women in the study did not experience any traumatic event, whereas nearly 44.7% had more than 2 (Supplementary Fig. 1). The participants who experienced 2 or more traumatic events were included in the high ACE group, while those with 0 or 1 were included in the low ACE group. Emotional violence, natural disaster and family conflict were the most frequent traumatic events reported by 35, 35 and 32% of the mothers respectively. None of the women in the study group was adopted or experienced war.

The two groups of mothers did not differ in terms of age at pregnancy, anthropometric measurements, the risk of PPD, as well as level of financial satisfaction. However, at 5 and 12 months of age, children from mothers in the high ACE group had significantly higher body weight and head circumference than those in the low ACE group (Supplementary Table 2).

### Association of maternal ACE with milk miRNAs

Milk RNA from women with extreme ELSQ scores (approximately top 25% lowest or highest) were used for sequencing; the very low ACE group (*n* = 14; mean ELSQ = 0.07 ± 0.27) and the very high ACE group (*n* = 13; mean ELSQ = 6.80 ± 1.20). The number of reads did not differ significantly between the groups with a mean total number of 25,889,422 reads in the very low ACE and 23,217,801 in the very high ACE group. MiRNA reads constituted 18 and 20% of the total reads in the very low ACE and very high ACE groups respectively (Supplementary Table 4). The 20 most abundant miRNAs in our samples were previously found to be the most expressed miRNAs in other studies of human breast milk ([21]) (Supplementary Fig. 2).

**Fig. 2:**
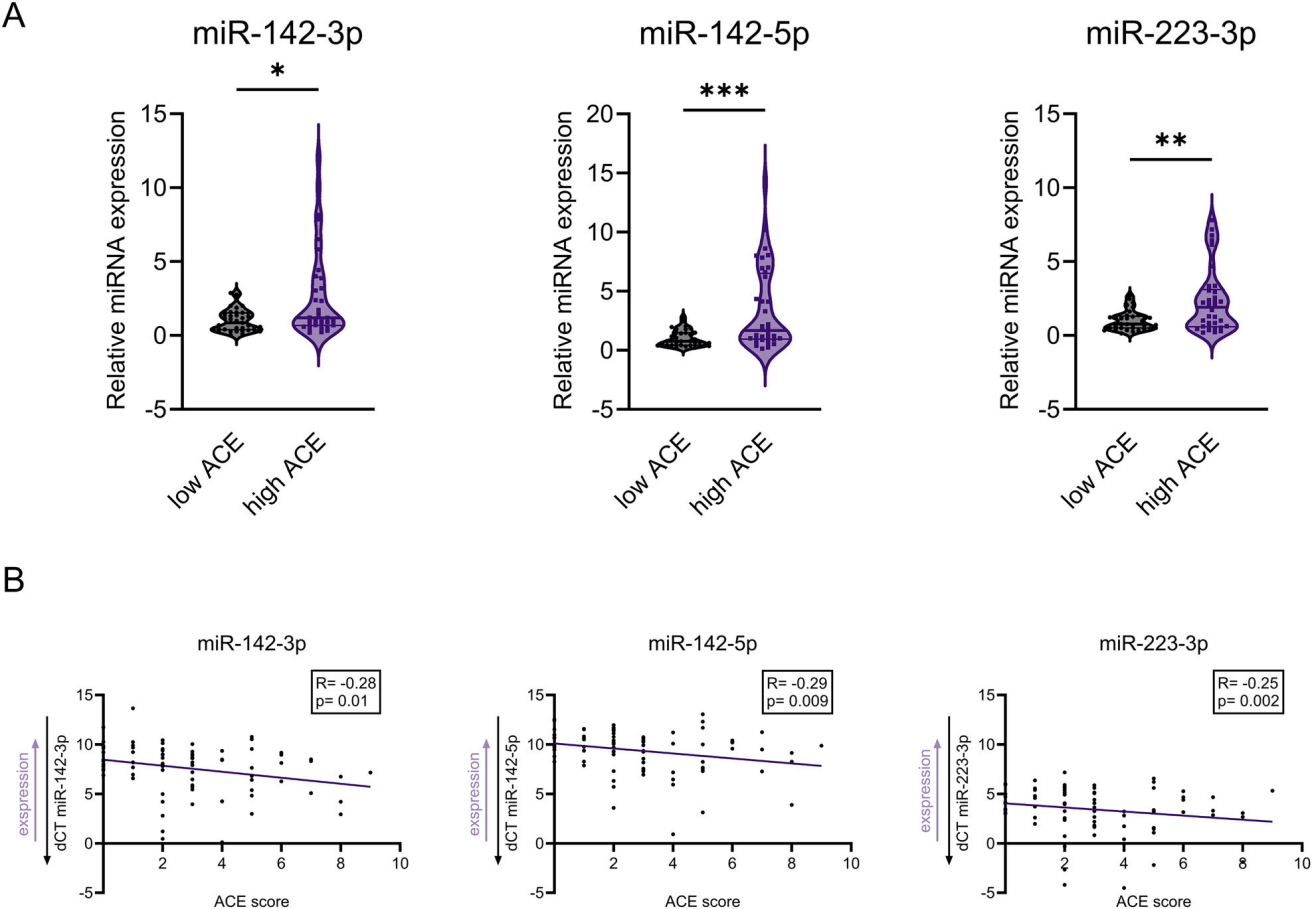
Effects of ACE on milk miRNAs. **A** miRNAs expression levels in the milk from low (*n* = 43) vs. high ACE groups (*n* = 40) assessed by qPCR assays. Violin plots include individual values supplemented with the group median and quartiles. Outliers removed based on ROUT Q = 1%. Mann-Whitney test, *p < 0.05; **p < 0.01; ***p < 0.001 **B** Spearman correlation between ACE score and miR-142-3p, miR-142-5p and miR-223-3p expression quantified via qPCR assays (decreasing dCT indicates increasing expression).

The principal component analysis (PCA) did not reveal a particular clustering within the very low ACE and very high ACE groups (Supplementary Fig. 3). However, in differential expression analysis, three miRNAs were found to be upregulated (p_adj_ < 0.1) in the very high ACE group compared to the very low ACE group: hsa-miR-142-3p (log^2^FoldChange = 2.15; p_adj_ = 0.015), hsa-miR-223-3p (log^2^FoldChange = 2.17; p_adj_ = 0.055), hsa-miR-142-5p (log^2^FoldChange = 2.19; p_adj_ = 0.082) (Fig. 2, Supplementary Table 5). The differentially expressed miRNAs were among the top 14% of all detected small RNAs as indicated by their base means.

Sequencing analysis of miRNAs expression in very low ACE and very high ACE groups was followed by confirmation with qPCR assay on all available samples. qPCRs were performed on 43 low ACE and 40 high ACE milk samples. A total of 20 samples were excluded from the analysis based on ambiguity of psychological data, unavailability of milk samples, low abundance or quality of the extracted RNA. However, the samples included for qPCR validation were still appropriate as per power analysis calculations (effect size between two independent groups = 0.25; power of the study = 90%; α = 0.05), which indicated a minimum *n* = 33 per group. Expression of all selected miRNA was significantly higher in the high ACE group compared to the low ACE group (miR-142-5p: fold change = 3.42, *p* = 0.0004; miR-223-3p: fold change = 2.35, *p* = 0.0094; miR-142-3p: fold change = 2.72, *p* = 0.0157) (Fig. 2A). Furthermore, the expression of miR-142-5p remained significantly elevated in the high ACE group when the samples were categorized into three instead of two groups based on ACE scores: low (0–1), medium (2), and high (≥3). Similarly, miR-142-3p showed a trend (*p* < 0.1) towards increased expression in the high ACE group (Supplementary Fig. 4). To further explore the relationship between maternal ACE and milk miRNAs, we performed correlation analyses across the full range of ACE scores. All three miRNAs — miR-142-3p, miR-142-5p, and miR-223-3p — showed a positive correlation with maternal ACE scores, indicating higher expression in the breast milk from women with increasing ACE score (Fig. 2B).

To ensure that these association were not confounded by a history of PPD, we compared milk miRNA expression in mothers with and without a PPD history and examined correlations between milk miRNAs and EPDS. Expression levels of miR-142-3p, miR-142-5p, and miR-223-3p were comparable between the two groups (Supplementary Fig. 5A). Additionally, no significant correlations were found between EPDS scores and expression of the miRNAs in the breast milk (Supplementary Fig. 5B).

### Association of maternal ACE with milk fatty acids

**Fig. 3:**
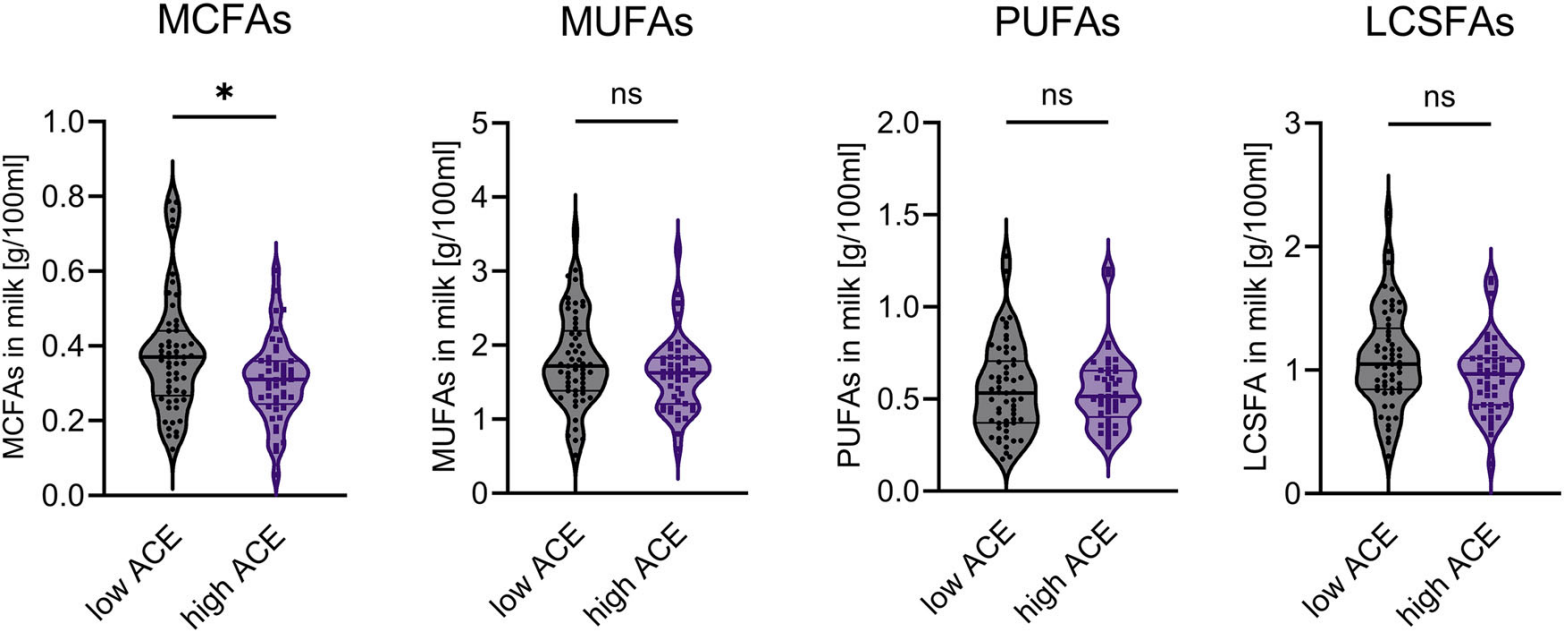
Comparison of FAs in the breast milk of women with high vs. low ACE. Violin plots include individual values supplemented with the group median and quartiles. Outliers removed based on ROUT Q = 1%. Mann-Whitney test, * *p* < 0.05, ns = *p* > 0.1.

The concentration of MCFAs was found to be significantly lower in the milk samples from the high ACE group vs. those from the low ACE group (*p* = 0.021; median in low ACE = 0.37 and in high ACE = 0.31). However, no significant differences were observed when the two groups were compared on LCSFAs, MUFAs, and PUFAs (*p* = 0.054; *p* = 0.140; *p* = 0.952, respectively) (Fig. 3). As the composition of fatty acids can also be influenced by the dietary habits of the mother, ANCOVA models were designed to control for maternal BMI and fat intake. The association of high ACE with lower milk MCFAs was confirmed even after adjusting for maternal fat intake, maternal BMI, as well as infant sex, on ANCOVA models (F = 5.48; *p* = 0.021) (Table 1). Neither the daily fat intake nor milk fat content differed between the high vs. low ACE groups (Supplementary Table 3).

**Table 1:** Result of ANCOVA models for middle chain fatty acids (MCFAs), polyunsaturated fatty acids (PUFAs), monounsaturated fatty acids (MUFAs) and long chain saturated fatty acids (LCSFAs).

	F	*p* value
Middle chain fatty acids (MCFAs)		
Maternal ACE (low-high)	5.48	**0.021**
Maternal BMI	2.85	0.095
Maternal fat intake	3.40	0.068
Infant sex (boys-girls)	0.00	0.998
Polyunsaturated fatty acids (PUFAs)		
Maternal trauma (low-high)	0.11	0.739
Maternal BMI	1.44	0.233
Maternal fat intake	2.74	0.101
Infant sex (boys-girls)	1.49	0.225
Monounsaturated (MUFAs)		
Maternal ACE (low-high)	2.98	0.088
Maternal BMI	1.01	0.318
Maternal fat intake	0.14	0.712
Infant sex (boys-girls)	0.26	0.611
Long chain saturated fatty acids (LCSFAs)		
Maternal ACE (low-high)	2.28	0.134
Maternal BMI	0.36	0.550
Maternal fat intake	0.79	0.389
Infant sex (boys-girls)	1.27	0.263

Significant effects are highlighted in bold.

### Correlation of specific infant behaviors with milk miRNAs

**Fig. 4:**
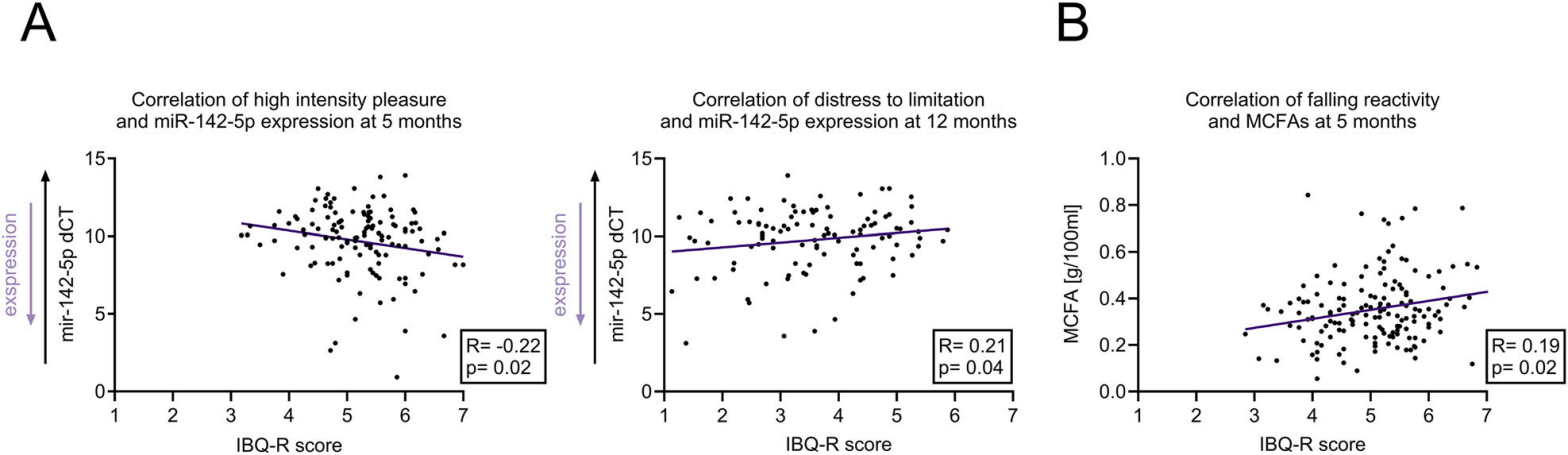
Correlations of milk miRNAs and FAs with infant temperament. **A** Spearman correlation between specific offspring behavioral traits with miR-142-5p expression at 5 and 12 months of age quantified via qPCR assays (decreasing dCT indicates increasing expression) **B** Correlation between falling reactivity at the age of 5 months with milk MCFAs.

We next ascertained correlations between the expression level of specific miRNAs as indicated by delta CT (dCT) values on qPCR in mothers’ milk with the temperamental scores of the infants. The dCT values of miR-142-5p showed a significant negative correlation (r = −0.215; *p* = 0.02) with high intensity pleasure in the infants, i.e., higher expression of milk miR-142-5p (lower dCT) correlated with higher scores on high intensity pleasure assessment in the infants. Similarly, milk miR-142-5p dCT was positively correlated (r = 0.212; *p* = 0.035) with infant’s distress to limitation at 12 months of age, i.e., lower expression of milk miR-142-5p (higher dCT) correlated with higher distress to limitation score in the infants (Table 2, Fig. 4A).

**Table 2:** Correlations between specific offspring temperamental traits at the age of 5 and 12 months with milk miRNAs quantified by qPCR assays (decreasing dCT indicates increasing expression).

Temperamental trait		miR-142-3p dCT	miR-142-5p dCT	miR-223-3p dCT
5 months of age				
Surgency/extraversion	Approach	0.05	−0.02	0.03
	Vocal Reactivity	0.07	−0.02	0.06
	High Intensity Pleasure	−0.18	−**0.22**	−0.05
	Activity Level	0.03	0.01	0.00
	Perceptual Sensitivity	0.02	0.08	0.05
	Smiling and Laughter	0.06	−0.01	0.11
Negative affectivity	Fear	0.00	0.03	0.02
	Sadness	0.00	0.02	0.00
	Distress to Limitations	0.03	0.01	0.04
	Falling Reactivity	0.01	0.08	0.05
Orienting/regulation	Duration of Orienting	−0.07	−0.06	0.05
	Soothability	0.13	0.05	0.18
	Cuddliness	0.04	−0.09	0.11
	Low Intensity Pleasure	−0.01	−0.08	0.06
12 months of age				
Surgency/extraversion	Approach	0.14	0.09	0.08
	Vocal Reactivity	−0.17	−0.15	−0.05
	High Intensity Pleasure	0.04	0.02	0.09
	Activity Level	0.03	0.00	0.13
	Perceptual Sensitivity	0.07	−0.02	−0.02
	Smiling and Laughter	−0.01	0.01	0.01
Negative affectivity	Fear	0.08	0.02	0.13
	Sadness	0.06	0.14	0.13
	Distress to Limitations	0.15	**0.21**	0.13
	Falling Reactivity	−0.12	−0.10	−0.13
Orienting/regulation	Duration of Orienting	−0.09	−0.08	0.05
	Soothability	0.08	0.08	0.16
	Cuddliness	0.00	−0.12	0.08
	Low Intensity Pleasure	−0.07	−0.12	0.00

Significant (*p* < 0.05) correlations are highlighted in bold.

### Correlation of specific infant behaviors with milk fatty acids

Similar to the correlations between specific infant behaviors and milk miRNAs, milk FAs were also found to be associated with specific infant behaviors. MCFAs in milk positively correlated with infant’s falling reactivity at 5 months (r = 0.186; *p* = 0.02) (Fig. 4B). Whereas infant’s high-intensity pleasure (r = −0.183; *p* = 0.035) and distress to limitation (r = −0.178, *p* = 0.042) at the age of 12 months showed negative correlations with milk MCFAs. Moreover, milk LCSFAs and MUFAs showed a negative correlation with activity levels of the infants (LCSFA: r = −0.18; *p* = 0.038; MUFA: r = −0.178; *p* = 0.04). Furthermore, milk LCSFAs and MUFAs were correlated with infant soothability at 12 months of age (LCSFA: R = 0.219; *p* = 0.011, MUFA: r = 0.181; *p* = 0.037). Finally, milk PUFAs levels showed a negative correlation with the infant activity level at the age of 12 months (r = 0.174; *p* = 0.045) (Table 3, Supplementary Fig. 6).

**Table 3:** Correlations between specific offspring temperamental traits at the age of 5 and 12 months and FAs in the milk.

Temperamental trait		MCFAs	LCSFAs	MUFAs	PUFAs
5 months of age					
Surgency/extraversion	Approach	0.00	−0.03	−0.03	−0.08
	Vocal Reactivity	0.04	0.06	0.05	0.01
	High Intensity Pleasure	−0.09	−0.05	−0.02	−0.03
	Activity Level	−0.14	−0.13	−0.12	−0.02
	Perceptual Sensitivity	0.08	0.07	0.05	−0.10
	Smiling and Laughter	0.04	0.04	0.03	−0.01
Negative affectivity	Fear	0.10	0.14	0.11	0.07
	Sadness	−0.11	−0.09	−0.01	−0.02
	Distress to Limitations	−0.05	−0.04	0.01	0.04
	Falling Reactivity	**0.19**	0.14	0.08	−0.10
Orienting/regulation	Duration of orienting	0.12	0.09	0.12	0.09
	Soothability	0.15	0.14	0.10	−0.07
	Cuddliness	0.03	−0.04	−0.07	−0.03
	Low Intensity Pleasure	0.08	0.09	0.07	0.05
12 months of age					
Surgency/extraversion	Approach	−0.07	−0.04	−0.05	0.06
	Vocal Reactivity	−0.14	−0.10	−0.12	−0.08
	High Intensity Pleasure	−**0.18**	−0.11	−0.08	0.03
	Activity Level	−0.13	−**0.18**	−**0.18**	−**0.17**
	Perceptual Sensitivity	0.01	0.08	0.10	0.08
	Smiling and Laughter	−0.08	−0.07	−0.08	−0.06
Negative affectivity	Fear	0.03	0.04	0.05	0.00
	Sadness	−0.05	−0.09	−0.05	−0.02
	Distress to Limitations	−**0.18**	−0.16	−0.15	−0.04
	Falling Reactivity	0.04	0.06	0.06	0.03
Orienting/regulation	Duration of orienting	0.01	0.01	0.03	−0.01
	Soothability	0.15	**0.22**	**0.18**	0.07
	Cuddliness	−0.02	−0.08	−0.10	−0.08
	Low Intensity Pleasure	0.01	0.02	0.03	0.03

Significant (*p* < 0.05) correlations are highlighted in bold.

## Discussion

The results of this study indicate differential regulation of specific miRNAs and FAs in human breast milk after exposure to ACE. Milk collected from mothers with high ACE showed significantly increased miR-142-3p, miR-142-5p, and miR-223-3p compared to the milk from mothers with low ACE. Notably, all three miRNAs increased in the milk with increasing ACE scores. Furthermore, MCFAs levels were found to be significantly lower in the milk from mothers with a history of high ACE compared to those from low ACE. Interestingly, high ACE-associated miR-142-5p and MCFAs both correlated with specific infant behaviors. Increased expression of miR-142-5p was associated with high intensity pleasure and distress to limitation, whereas milk MCFAs correlated with falling reactivity. To the best of our knowledge, our study is the first demonstration of specific miRNA and FA signatures of ACE in maternal milk in humans. Furthermore, correlation of miR-142-5p and MCFAs with maternal assessment of infant temperament represents a plausible, albeit debatable, role for milk miRNAs and FAs in regulation of early temperamental development of infants. Finally, these results propose milk miRNAs and FAs as potential predictors of early intergenerational behavioral effects of ACE in humans.

A potential mediating role of milk in the intergenerational transmission of the effects of ACE is suggested by emerging evidence indicating both direct and indirect routes of mediation. For instance, ACE could indirectly affect the cognitive and behavioral development of the child via milk through alteration of maternal lactation behaviors. Indeed, there is evidence that perinatal psychopathologies, such as PPD are associated with lower rates of breast-feeding initiation and shorter duration [49–52]. However, in our study, the feeding episodes per 24 h did not significantly differ between the high ACE and low ACE groups with an average of 11 episodes in both the groups. Furthermore, the prevalence of postpartum depression did not differ between the two groups (Supplementary Table 2). However, it is important to maintain a nuanced and cautious perspective before attributing causality to the observed changes in miRNAs and fatty acids in the breast milk as being directly responsible for the associated infant behavioral outcomes. Multiple confounding factors, including genetic predispositions, maternal cortisol signaling, maternal alcohol or drug abuse, maternal socioeconomic status, birth and post-natal environments, as well as maternal personality types and pre-natal experiences can contribute to infant behavioral patterns [53]. Furthermore, not only maternal but also paternal behaviors can impact offspring temperament. Fathers may contribute to offspring development directly through their involvement in caregiving and emotional support, especially in species with biparental parenting like humans. Paternal behaviors can also indirectly influence infant temperament by affecting maternal stress levels, which in turn shape mother-infant interactions [54, 55].

Breast milk is a rich source of miRNAs with more than 1400 miRNAs expressed at detectable levels [56, 57]. Importantly, miRNAs that are abundantly expressed in the milk have pertinent regulatory roles in lipid and glucose metabolism, gut maturation, neurogenesis and immune system [58, 59]. There is substantial evidence, from studies in several mammalian models, that miRNAs in the breast milk are attenuated by maternal health factors and may impact offspring development and behavior [60–64]. However, the mechanisms underlying these effects remain disputed. As per the ‘nutritional hypothesis’, milk ncRNAs are degraded in the digestive tract of the offspring and release nucleotides that serve local nutritional requirements for the offspring intestinal cells or microbiome [65]. However, accumulating evidence supports that milk miRNAs are able to at least partially evade degradation in the digestive tract because of their packaging in exosomes. Subsequently, milk miRNAs are stably absorbed in the offspring blood, and regulate gene functions in different offspring organs [59, 66, 67]. Studies on milk exosomes labeled with fluorophores or fluorescent fusion proteins demonstrated their accumulation in the offspring liver, spleen, and brain after suckling in mice and pigs [68]. Furthermore, oral administration of bovine milk exosomes transfected with fluorophore-labeled synthetic miRNAs led to accumulation and tissue-specific distribution of miRNAs in the intestinal mucosa, spleen, liver, heart and brain of mice [68]. Another evidence for the functionality of milk miRNAs comes from a mouse study that showed corresponding changes in the milk ncRNAs and duodenal proteome of the suckling pups after exposure to high-fat diet in the mothers [69].

The miRNAs found to be altered after high ACE in the mothers’ milk in this study have been previously associated with regulation of stress-related behaviors. Exposure to single prolonged stress in rats was shown to increase hippocampal miR-142-5p, which resulted in anxiety-like behavior, memory deficits, and aberrant plasticity [70]. Notably, inhibition of miR-142-5p in the amygdala or hippocampus were both associated with decreased behavioral deficits after exposure to single prolonged stress in rats [70, 71]. Similarly, peripheral levels of miR-223-3p have been associated with therapeutic responses in patients with treatment-resistant depression [72]. Furthermore, both miR-142 and miR-223 are abundantly expressed in the milk and have been associated with mammary gland health and functions. miR-142-3p was previously shown to regulate development of milk lobules and ducts in the murine mammary glands with functional effects on the amount of milk produced [73]. Similarly, miR-223 was shown to regulate immune interactions of mammary epithelial cells with critical implications for mastitis [74].

Furthermore, several previous studies have revealed the influence of milk FAs composition on the cognitive and temperamental indicators of the offspring. However, the focus of most of these studies has been on PUFAs, such as docosahexaenoic acid (DHA) and eicosapentaenoic acid (EPA). In a study evaluating maternal DHA supplementation, children who consumed DHA-supplemented milk demonstrated a significantly higher mental development index at 30 months of age compared with those who did not [75]. Furthermore, a more recent study showed less negative affectivity in infants breastfed with milk richer in n-3 PUFAs [76]. To the best of our knowledge, the specific association between milk MCFAs or miR-142 with offspring temperamental indices has not been explored previously either in humans or rodents.

Because of their abundant expression in human milk, as well as the observed correlations with offspring behaviors, milk miR-142-5p and MCFAs deserve further exploration as early predictors of the intergenerational effects of ACE in humans. Notably, the overall comparison of infant temperament between the children born to mothers with high ACE vs. low ACE from this cohort did not reveal any significant differences in previous studies (Apanasewicz A et al. 2020, 2023). However, a clear correlation was observed between the milk miR-142-5p and MCFAs and certain offspring temperamental indicators of affect. Interestingly, all ACE-associated miRNAs in this study; miR-142-3p, miR-142-5p, and miR-223-3p are known to regulate lipid metabolism [77, 78]. Hence, concomitant changes in FAs and miR-142-3p, miR-142-5p, and miR-223-3p in the mothers’ milk could reflect broader interlinked aberrations in lipid metabolism. Furthermore, the ACE-induced changes in milk miRNAs and FAs may also impact offspring temperament via altering the gut microbiome of the offspring. This is supported by several human studies where overall microbiome diversity, as well as specific microbial species have been associated with parameters of surgency, extraversion, and negativity affectivity of the children [79–82].

In conclusion, this study establishes a significant association between maternal ACE and alterations in the miRNAs and FAs content of breast milk, which may serve as early predictors of temperamental trajectories in the offspring. However, it is critical to acknowledge that the effects of maternal ACE on offspring are likely mediated through multiple intergenerational pathways. These include epigenetic modifications in the oocyte, microbiome transmission during vaginal delivery, and maternal behaviors both during and after pregnancy, in addition to lactation. Additionally, the present study did not account for paternal influences, which could represent an important source of confounding and warrant further investigations.

To establish the biological plausibility of the observed associations and to determine any causal mechanisms, we are initiating mechanistic studies in a mouse model of early postnatal trauma. These include cross-fostering experiments and evaluation of specific miRNAs. Cross-fostering will be conducted within 12 h of birth between litters of dams exposed to MSUS and control dams, with careful behavioral monitoring including maternal grooming. Milk samples will be collected on postnatal day 7 and 14 and analyzed for miRNAs and FAs composition using unbiased transcriptomic and metabolomic approaches. The offspring will then be followed till late adulthood and serially phenotyped for behavioral and metabolic alterations. These studies aim to elucidate the mechanistic role of breast milk constituents in shaping offspring behavioral outcomes, building upon the intriguing observations in our human cohort.

Furthermore, we continue to monitor the physical and mental health parameters of the children in our cohort, now aged between 6 and 8 years. In addition to yielding critical associations, this longitudinal follow-up will help consolidate the observed correlations between milk miR-142-5p and MCFAs and the temperamental variations in the offspring, thereby minimizing potential biases introduced by maternal subjective behavioral evaluations. Additionally, we have collected blood samples from the children, which are currently being analyzed to compare miRNA changes with those observed in maternal blood and milk. Future human studies may also incorporate broader epigenetic assessments and neuroimaging correlates to deepen our understanding. Notably, even before these studies are completed, the distinct miRNA and FA signatures of ACE in human breast milk in the current study suggest their potential as biomarkers for ACE and predictors of intergenerational transmission of ACE effects in humans.

## Supplementary information

File containing all supplementary material

## Data Availability

The data are not publicly available but anonymized versions of the data can be shared for research purposes only upon reasonable request to the corresponding author.
