# Supplementary material for: Differential microRNAs and metabolites in the breast milk of mothers with adverse childhood experiences
Source: Transl Psychiatry. 2025 Oct 6;15:367. doi: 10.1038/s41398-025-03491-4 (PMC12501080; doi:10.1038/s41398-025-03491-4)
Supplement: Supplementary file 1 — File containing all supplementary material [file 41398_2025_3491_MOESM1_ESM.docx]

**Supplemental Material**

**Supplementary Table 1.** Categorization of fatty acids.

| **Category** | **Symbol** | **Common name** |
| --- | --- | --- |
| MCFA | C10:0 | capric acid |
|  | C12:0 | lauric acid |
|  | C14:0 | myristic acid |
| LCSFA | C16:0, C18:0 | palmitic acid |
|  |  | stearic acid |
| MUFA | C16:1 | palmitoleic acid |
|  | C18:1 | oleic acid |
|  | C20:1 | eicosanoid acid |
| PUFA | C18:2 | linoleic acid |
|  | C18:3 | α-linoleic acid |
|  | C20:4 | arachidonic acid |
|  | C20:5 | eicosapentaenoic acid |
|  | C22:6 | docosahexaenoic acid |

**Supplementary Table 2.** Clinical and demographic characteristics of the mother-child dyads. Significant differences between the high ACE vs. low ACE groups are highlighted in bold and asterisked.

| **Mothers’ demographics, pregnancy parameters, and birth outcomes** | | | | | |
| --- | --- | --- | --- | --- | --- |
|  |  |  |  |  |  |
|  | **All participants (N = 158)** | **Low ACE (N = 57)** | **High ACE (N = 46)** | **p-value** |  |
|  | **Mean (SD)** | **Mean (SD)** | **Mean (SD)** |  |  |
| ELSQ score | 2.82 (2.30) | 1.18 (0.83) | 4.85 (1.89) | **< 0.001*** |  |
| Maternal age [years] | 30.64 (3.76) | 31.17 (3.84) | 29.74 (3.39) | 0.053 |  |
| Maternal BMI before pregnancy [kg/m^2^] | 22.61 (3.63) | 22.31 (2.92) | 23.22 (4.06) | 0.097 |  |
| Infant sex [boys%] | 53.16 | 54.38 | 58.69 | 0.665 |  |
| Gestational age [weeks] | 39.85 (1.38) | 39.72 (1.37) | 40.04 (1.51) | 0.261 |  |
| Infant body length [cm] | 54.70 (2.84) | 54.29 (3.06) | 55.13 (2.82) | 0.153 |  |
| Infant weight [g] | 3505 (463) | 3410 (442) | 3584 (470) | 0.058 |  |
| Infant head circumference [cm] | 34.05 (1.62) | 34 (1.47) | 34.07 (2.03) | 0.852 |  |
| EPDS score | 6.92 (4.72) | 6.67 (4.61) | 7.37 (4.97) | 0.447 |  |
| **Infant characteristics at 5 months of age** | | | | | |
|  |  |  |  |  |  |
|  | **All participants (N = 158)** | **Low ACE (N = 57)** | **High ACE (N = 46)** | **p-value** |  |
|  | **Mean (SD)** | **Mean (SD)** | **Mean (SD)** |  |  |
| Maternal BMI [kg/m^2^] | 23.20 (3.87) | 22.48 (3.18) | 23.49 (4.00) | 0.159 |  |
| Infant age [months] | 4.78 (0.58) | 4.80 (0.61) | 4.71 (0.54) | 0.443 |  |
| Infant body length [cm] | 66.36 (3.13) | 65.77 (2.88) | 66.38 (3.25) | 0.321 |  |
| Infant weight [g] | 7089 (922) | 6838 (785) | 7364 (1002) | **0.008*** |  |
| Infant head circumference [cm] | 42.10 (1.48) | 41.76 (1.35) | 42.40 (1.50) | **0.029*** |  |

| **Infant characteristics at 12 months of age** | | | | |  |
| --- | --- | --- | --- | --- | --- |
|  |  |  |  |  | |
|  | **All participants (N = 144)** | **Low ACE (N = 55)** | **High ACE (N = 44)** | **p-value** | |
|  | **Mean (SD)** | **Mean (SD)** | **Mean (SD)** |  |  |
| Maternal BMI [kg/m^2^] | 22.41 (3.81) | 21.48 (3.26) | 22.78 (4.34) | 0.102 | |
| Infant age [months] | 12.42 (0.71) | 12.36 (0.61) | 12.34 (0.84) | 0.875 | |
| Infant body length [cm] | 76.40 (3.07) | 75.55 (2.67) | 76.76 (3.71) | 0.074 | |
| Infant weight [g] | 9549 (1027) | 9180 (986) | 9842 (1025) | **0.002*** | |
| Infant head circumference [cm] | 46.00 (1.56) | 45.57 (1.78) | 46.40 (1.50) | **0.014*** | |

**Supplementary Table 3.** Comparison of fat intake and milk fat content between mothers with low and high ACE.

| **Mothers’ daily fat intake (mean from 3 days) and milk fat content** | | | | | | | |
| --- | --- | --- | --- | --- | --- | --- | --- |
|  |  | |  | |  | |  |
|  | **All participants (N = 158)** | **Low ACE (N = 57)** | | **High ACE (N = 46)** | | **p-value** | |
|  | **Mean (SD)** | **Mean (SD)** | | **Mean (SD)** | |  | |
| Maternal fat intake [g] | 79.40 (46.43) | 74.99 (32.10) | | 78.12 (25.80) | | 0.248 | |
| Milk fat [g/100ml] | 4.22 (1.39) | 4.36 (1.51) | | 3.89 (1.17) | | 0.089 | |

**Supplementary Table 4.** Comparison of the very high vs. low ACE groups included in the small RNA sequencing analysis.

| **RNA sequencing analysis** | | |
| --- | --- | --- |
|  | **Very low ACE  n=14** | **Very high ACE  n=13** |
| ELSQ (SD) | 0.071 (0.27) | 6.80 (1.20) |
| Total reads; millions (SD) | 25.89 (6.17) | 23.22 (6.21) |
| miRNA reads; millions (SD) | 4.57 (2.83) | 4.53 (4.53) |
| miRNA reads; % raw reads | 18 | 20 |

**Supplementary Table 5.** miRNAs differentially upregulated in the breast milk of mothers with high vs. low ACE.

| **miRNA** | **baseMean** | **log2FoldChange** | **p-value** | **padj** |
| --- | --- | --- | --- | --- |
| hsa-miR-146b-5p |  | 0.34 | >0.999 |  |
| hsa-miR-142-3p | 350.2117211 | 2.15 | <0.001 | 0.015 |
| hsa-miR-142-5p | 157.8526605 | 2.17 | <0.001 | 0.056 |
| hsa-miR-223-3p | 2162.150883 | 2.19 | <0.001 | 0.082 |

**Supplementary Figure 1.** Top miRNAs present in the milk samples as per small RNA sequencing.


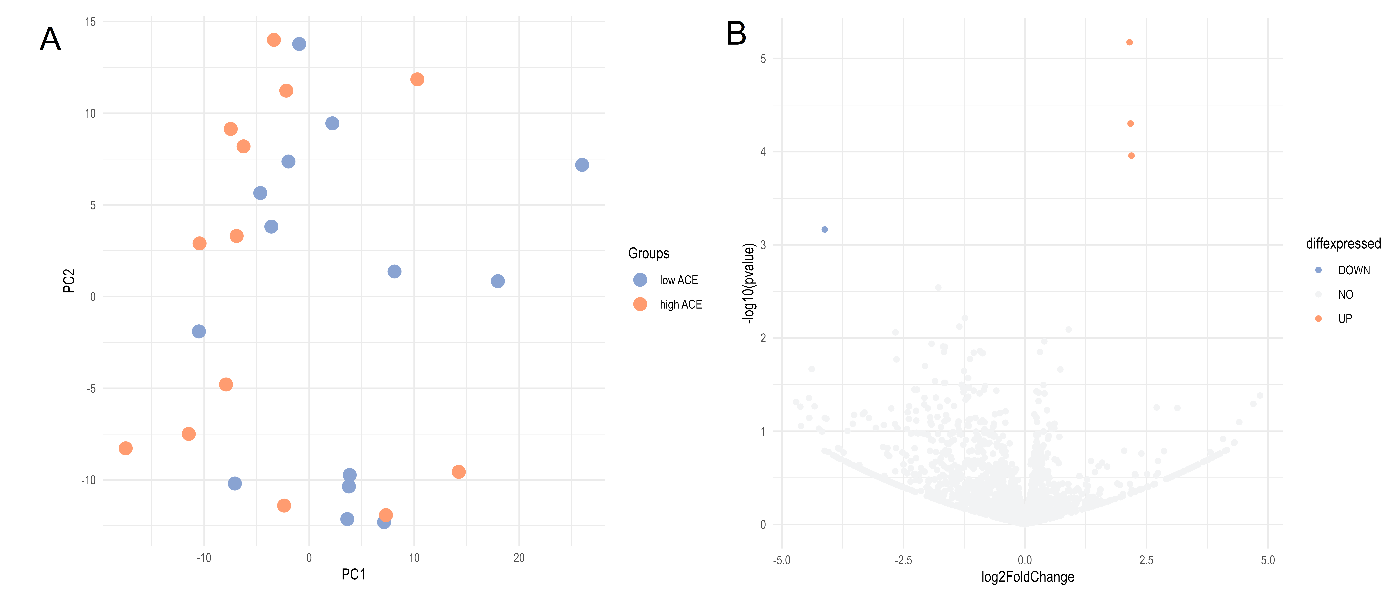


**Supplementary Figure 2.** PCA and volcano plot with miRNAs (A) The principal component analysis (PCA) scatter plot of miRNA expression in the 27 samples. The percentages on each axis represent the percentages of variation explained by the principal components. (B) Volcano plot showing miRNAs differentially expressed in the milk from high ACE.


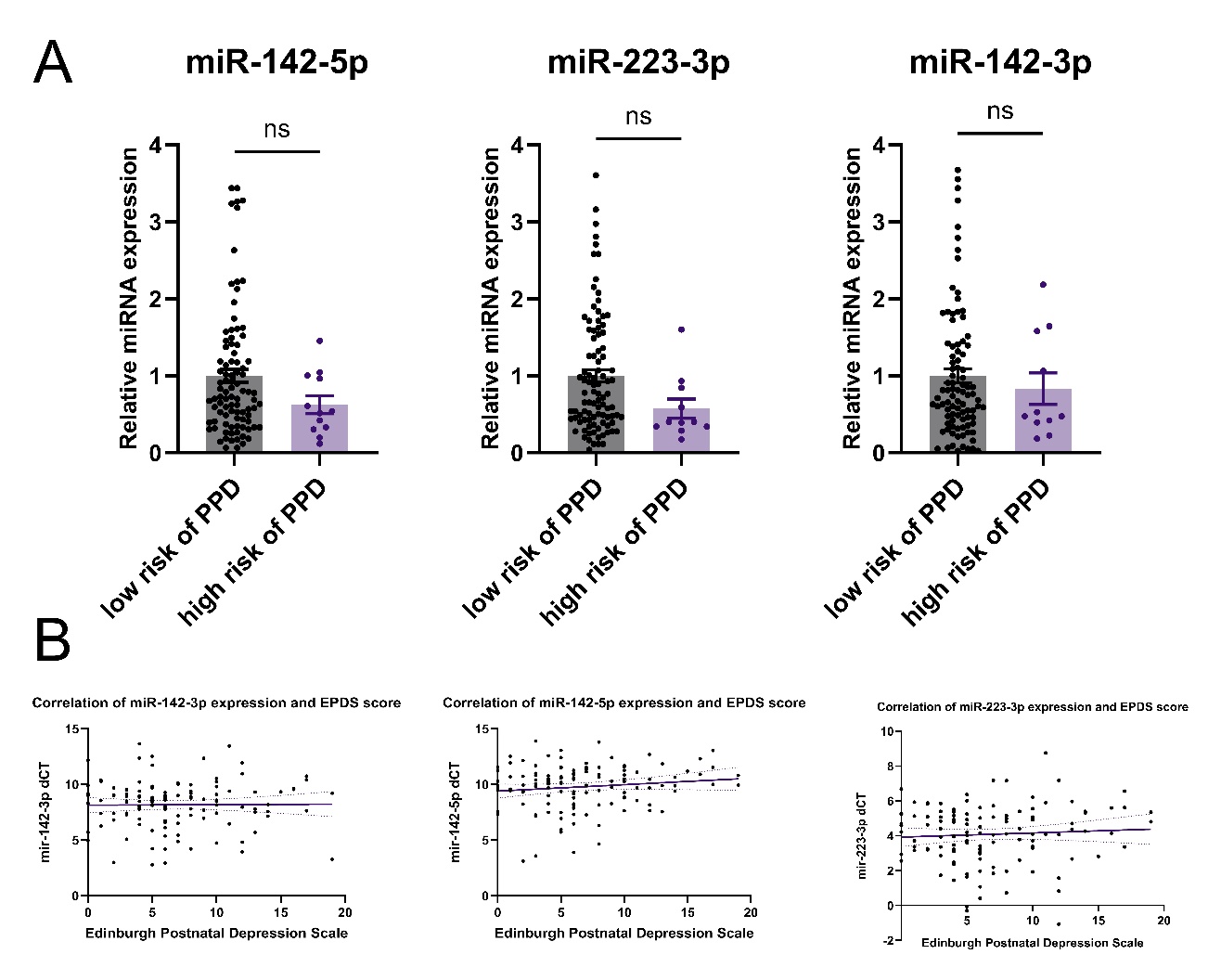


**Supplementary Figure 3.** miRNA expression level and risk of postpartum depression (A) Bar graphs with individual values of miR-142-3p, miR-142-5p and miR-223-3p expression level in mothers with low vs. high risk of postpartum depression (PPD) based on EPDS (low <13, high ≥13). (B) Spearman correlation between EPDS score and miR-142-3p, miR-142-5p and miR-223-3p expression level.


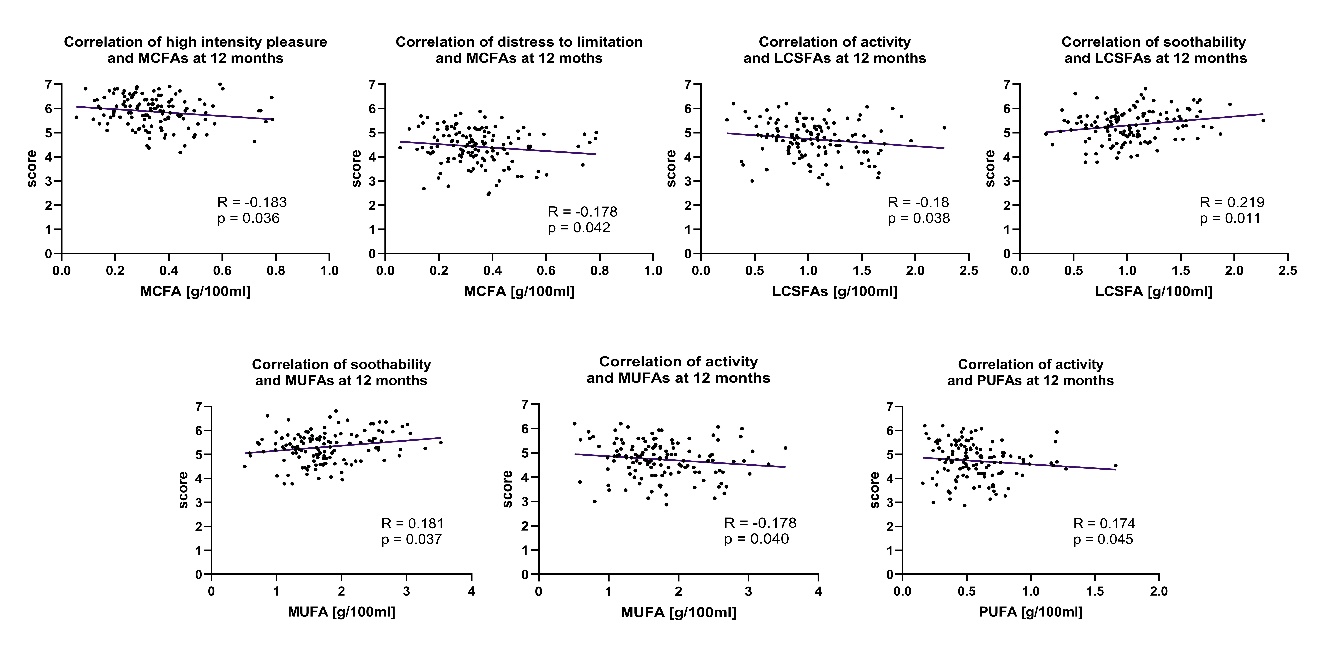


**Supplementary Figure 4.** Correlation of specific offspring behavioral traits at 12 months of age with fatty acids in milk.

**
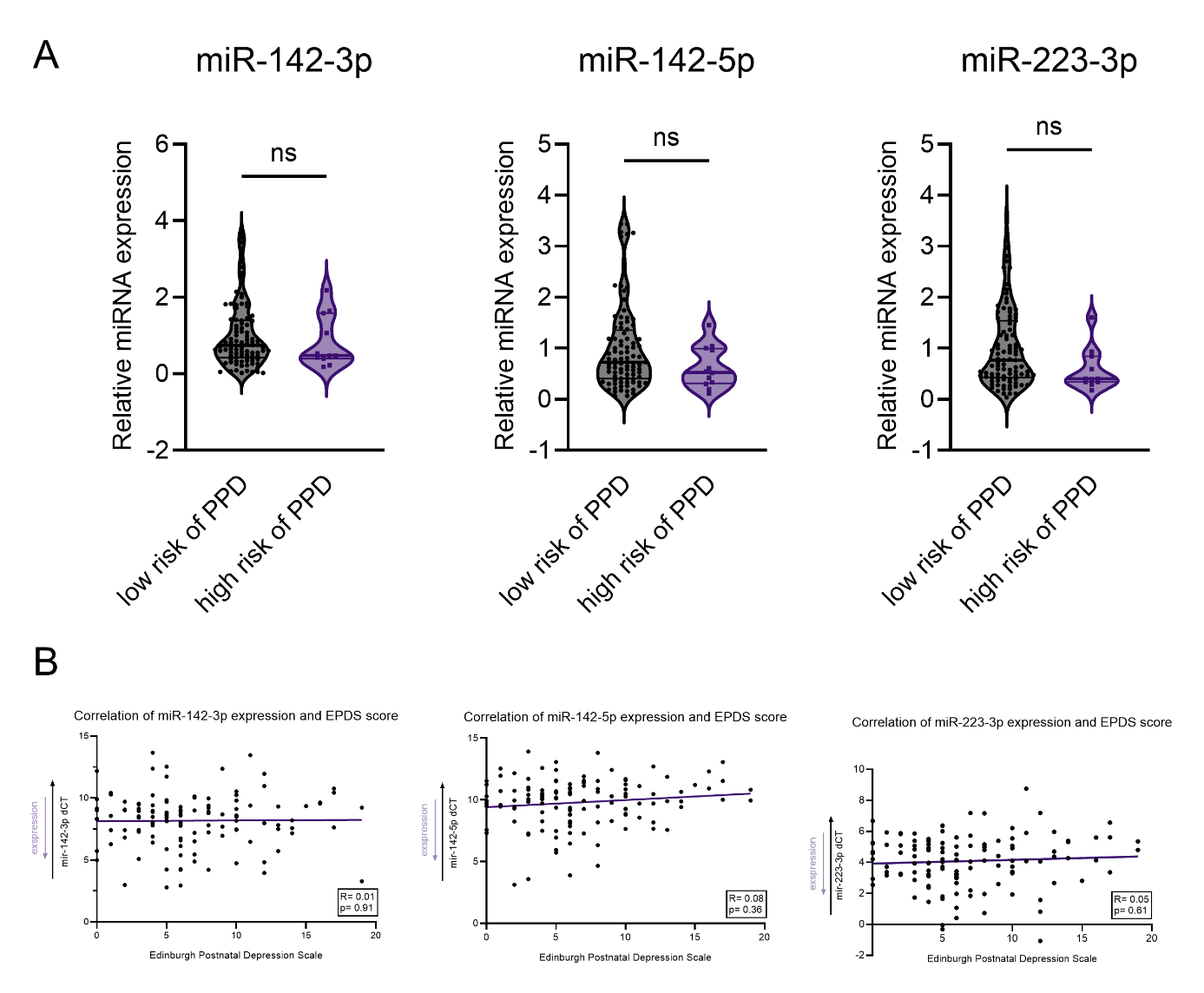
Supplementary Figure 5.** miRNA expression level and risk of postpartum depression (A) Violin plots with individual values of miR-142-3p, miR-142-5p and miR-223-3p expression in mothers with low vs. high risk of postpartum depression (PPD) based on EPDS (low <13, high ≥13) Outliers removed based on ROUT Q=1%. Mann-Whitney test, * p<0.05; ns= p>0.1. (B) Spearman correlation between EPDS score and miR-142-3p, miR-142-5p and miR-223-3p expression level quantified by qPCR assays (decreasing dCT indicates increasing expression).


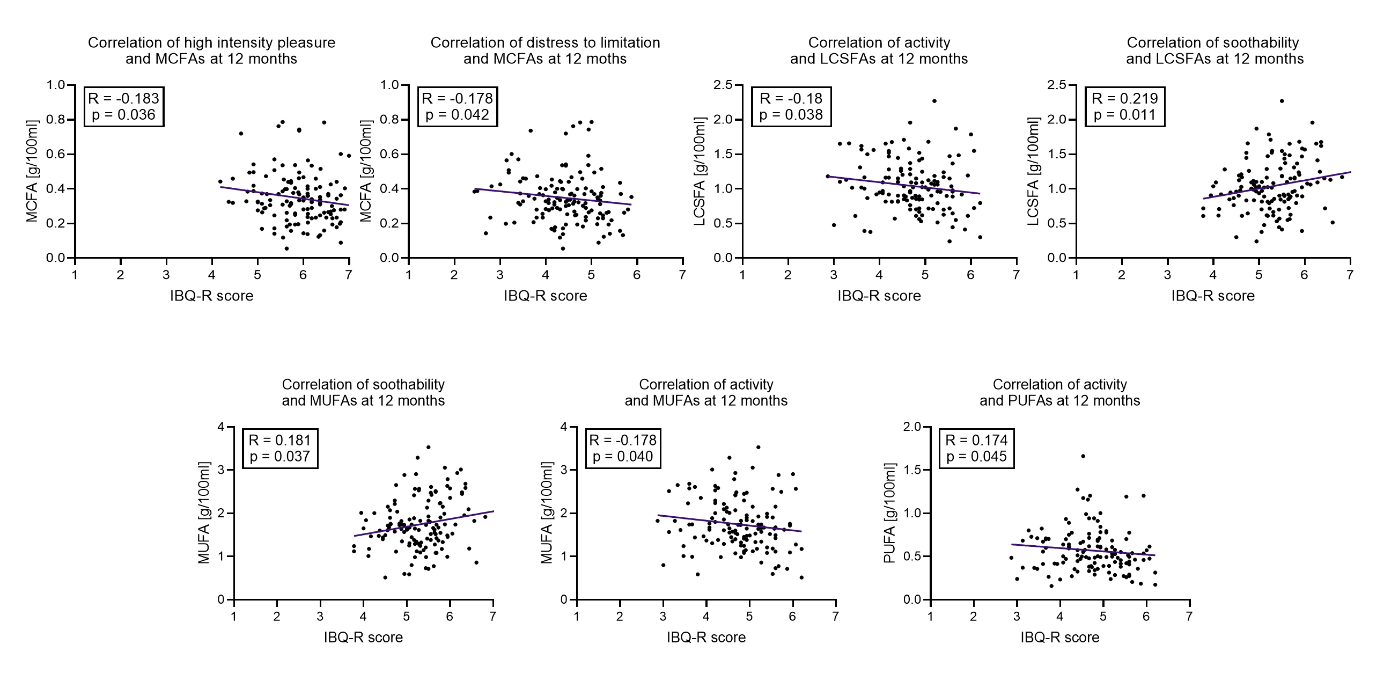
**Supplementary Figure 6.** Correlation of specific offspring behavioural traits at 12 months of age with fatty acids in milk.
